# Hormonal Treatment for Severe Hydronephrosis Caused by Bladder Endometriosis

**DOI:** 10.1155/2014/891295

**Published:** 2014-11-18

**Authors:** Erkan Efe, Murat Bakacak, Salih Serin, Eyüp Kolus, Önder Ercan, Sefa Resim

**Affiliations:** ^1^Department of Urology, Kahramanmaraş Sütçü İmam School of Medicine, Turkey; ^2^Department of Obstetrics and Gynecology, Kahramanmaraş Sütçü İmam School of Medicine, Kahramanmaras, Turkey

## Abstract

The incidence of endometriosis cases involving the urinary system has recently increased, and the bladder is a specific zone where endometriosis is most commonly seen in the urinary system. In the case presented here, a patient presented to the emergency department with the complaint of side pain and was examined and diagnosed with severe hydronephrosis and bladder endometriosis was determined in the etiology. After the patient was pathologically diagnosed, Levonorgestrel-Releasing Intrauterine System (LNG-IUS) was administered to the uterine cavity. At the 12-month follow-up, endometriosis was not observed in the cystoscopy and symptoms had completely regressed. Hydronephrosis may be observed after exposure of the ureter, and silent renal function loss may develop in patients suffering from endometriosis with bladder involvement. For patients with moderate or severe hydronephrosis associated with bladder endometriosis, LNG-IUS application may be separately and successfully used after conservative surgery.

## 1. Introduction

Endometriosis is defined as the appearance of the endometrial gland and stroma outside the uterus. Ectopic endometrial tissues are generally detected in the pelvis, although they may be observed anywhere in the body. Benign endometriosis is a chronic and estrogen-dependent disease, which may classically trigger certain symptoms such as pelvic pain, severe dysmenorrhea, dyspareunia, and infertility [1, 2]. On the other hand, deep infiltrating endometriosis (DIE) generally influences uterosacral ligaments, the rectovaginal region, posterior vaginal wall, intestines, and urinary system [3, 4].

The incidence of endometriosis cases involving the urinary system has recently increased, with reported rates of approximately 0.3–12% of all patients diagnosed with endometriosis [4–11]. The bladder is a specific zone of the urinary system where endometriosis is most commonly seen and bladder involvement is seen in approximately 85% of urinary system involvement endometriosis cases. To date, 350 cases have been defined in the literature [11]. In bladder endometriosis, the symptoms of the patient may vary according to the localization and dimensions of the lesion [11–13]. Basically, the most commonly encountered symptoms are acute urethral syndrome, tenesmus, burning sensation in the urinary tract, dysuria, suprapubic sensitivity, and pain accompanied by thamuria [11–17]. Menouria during the menstrual period is a symptom which is not encountered as commonly as acute urethral syndrome and which is seen in only 20–25% of the patients with mucosal involvement [17].

In this paper, it was aimed to review literature in conjunction with analysis of a case where a patient presented to the emergency department with the complaint of side pain and was diagnosed with severe hydronephrosis and bladder endometriosis was determined in the etiology. Successful treatment was applied using the Levonorgestrel-Releasing Intrauterine System (LNG-IUS).

## 2. Case

**Figure 1:**
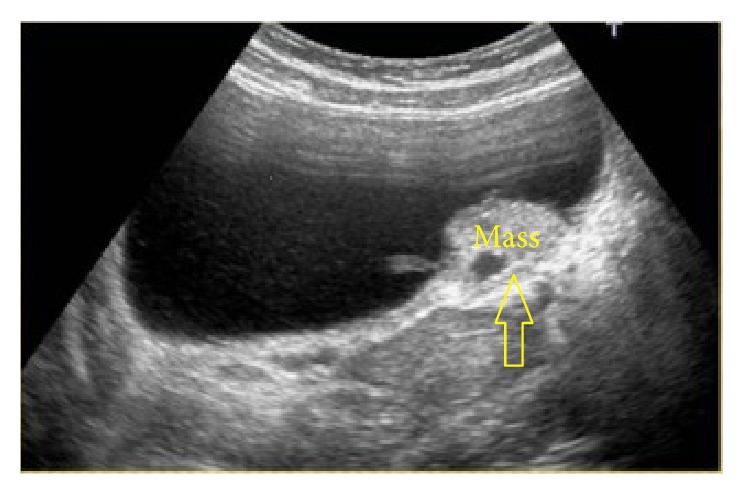
Urinary system ultrasonography showing a massive lesion of 3 × 4 cm extending into the bladder on the left side wall of the bladder.

**Figure 2:**
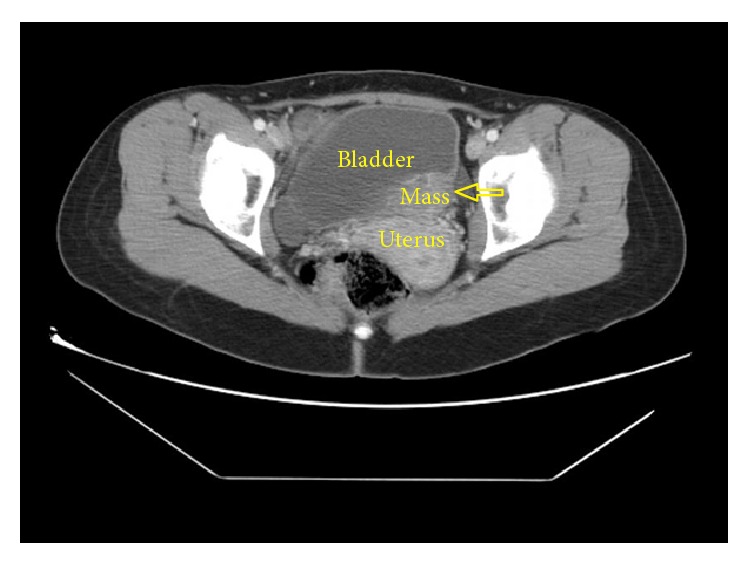
Abdominal contrast Computed Tomography showing a massive lesion of 3 × 4 cm extending into the bladder on the left ureter orifice on the left side wall of the bladder.

**Figure 3:**
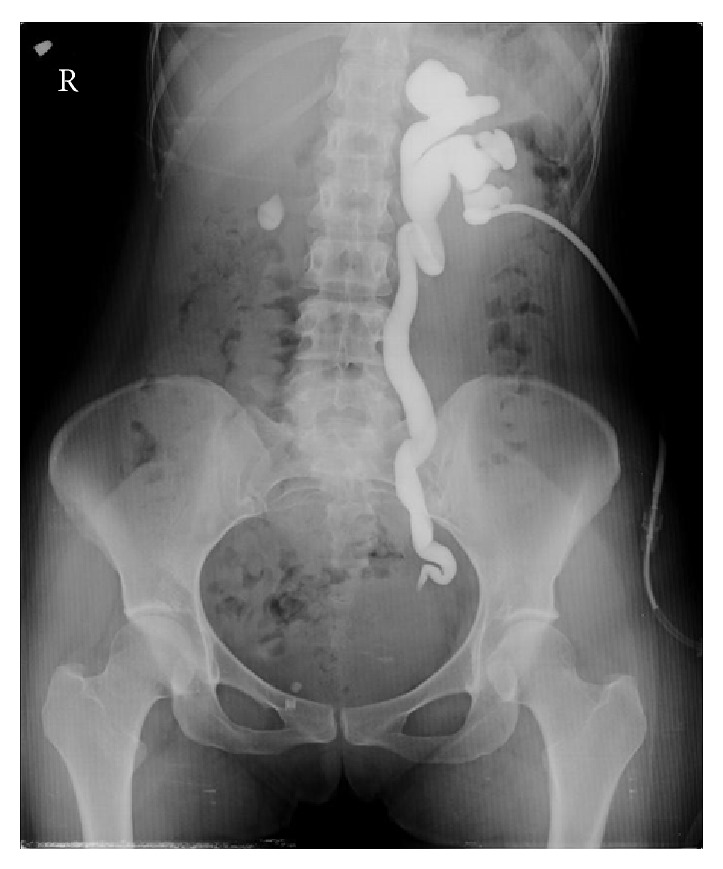
The anterograde pyelography was taken through the nephrostomy catheter and the contrast material was not transmitted to the bladder in the ureterovesical junction.

**Figure 4:**
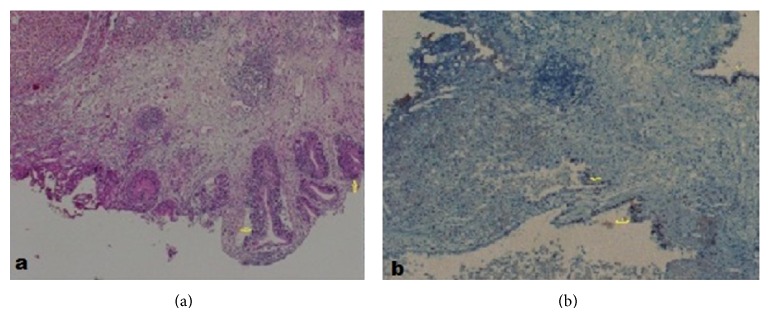
(a) Photomicrograph showing the hemosiderin-laden macrophages in the bladder wall. (Haematoxylin and eosin staining, original magnification ×40.) (b) Photomicrograph showing the estrogen-containing epithelial cells in the bladder wall. (Immunohistochemical staining, original magnification ×40.)

A 29-year-old female presented to the emergency department with the complaint of left side pain. Sensitivity in the left costovertebral region was detected during physical examination. No kind of operation or chronic disease was recorded in the medical history and family history of the patient. Creatinine value was found to be 1.37 ng/dL in the blood biochemistry examination. In the urinary system ultrasonography (USG), it was observed that the right kidney was atrophic, that the left ureter was dilated as far as the ureterovesical junction, that there was grade III hydronephrosis in the left kidney, and that there was a massive lesion of 3 × 4 cm extending into the bladder on the left side wall of the bladder (Figure 1). In each abdominal contrast Computed Tomography (CT) scan of the patient, it was seen that the left ureter and renal pelvis were severely dilated and that there was a massive lesion of 3 × 4 cm extending into the bladder on the left ureter orifice on the left side wall of the bladder (Figure 2). In the medical history of the patient, it was recorded that the patient had complaints of hematuria and severe pelvic pain which had been ongoing for 4-5 menstrual cycles. On transvaginal USG, a mass of 4 × 4 cm of cystic appearance was detected in simple form in the left ovary. In cystoscopy, the left orifice could not be seen although the right orifice was monitored. A red-colored solid lesion in the papillary structure was observed in the left area conforming to the symmetry of the right orifice. The lesion was partially resected by resectoscope. The resected material was sent to the pathology laboratory for histological examination. Percutaneous nephrostomy was attached to the left kidney under USG guidance. Contrast material was administered to the patient through the nephrostomy catheter, and anterograde pyelography was taken. It was observed that the contrast material was not transmitted to the bladder in the ureterovesical junction (Figure 3). At follow-up examinations, within 3 days, the hydronephrosis in the left kidney had receded and the serum creatinine value was determined to have returned to normal limits. The nephrostomy catheter was removed on the third day as renal functions had returned to normal and the hydronephrosis had receded. The final pathology result of the resected material was reported as “endometriosis” (Figure 4). The patient was referred to the gynaecology clinic. The tumor markers of the patient were determined as Ca 125: 92.2 *μ*/mL (0–35 *μ*/mL) and Ca 19.9: 138.4 *μ*/mL (0–37 *μ*/mL). Hormonal treatment was planned to be administered to the patient by the gynaecology and obstetrics department. During the surgery, the Levonorgestrel-Releasing Intrauterine System (Mirena) was inserted into the patient. After the application of LNG-IUS, while there was no reduction in the tumour markers at 3 months, when the tumour markers were evaluated at 6 months, they were seen to have receded to normal reference values. At the end of 12 months, the mass had completely recovered and the symptoms had disappeared.

## 3. Discussion

Endometriosis is seen in 3–10% of reproductive age women with complaints of pelvic pain [18]. DIE has been defined as the implantation of endometrial stroma and glandural epithelium outside the endometrial cavity and their penetration deeper than 5 mm into retroperitoneal regions and the surfaces of pelvic organs [19]. DIE may involve the posterior fornix, uterosacral ligament, rectum, vagina, and urinary system outside the uterus [4].

Strong data are not available regarding the prevalence of urinary system endometriosis. It is estimated that urinary system endometriosis constitutes 1-2% of all endometriosis cases [20, 21]. The bladder is affected by 85% of patients suffering from endometriosis involving the urinary system. The current preference for the treatment of the patients with bladder involvement is surgery, including transurethral resection or partial cystectomy followed by hormonal treatment [22].

Hydronephrosis may be observed after the exposure of the ureter, and silent renal function loss may develop in patients suffering from endometriosis in which bladder involvement is seen. Therefore, those patients should be closely monitored with renal function tests [14, 23]. Ultrasonography is the first method that should be applied, since it can be easily applied to the patient through abdominal, transvaginal, or transrectal routes depending on the complaints in the diagnosis of rare endometriosis cases such as bladder endometriosis, it is not an expensive method, and it does not cause radiation exposure [23–28]. Moreover, examination of urinary cytology plays an important role in the differential diagnosis of this disease from bladder cancer in the above-mentioned patients [12, 17].

Since the cells of endometriosis include estrogen and progesterone receptors, the disease responds to hormonal treatment [29, 30]. The objective of numerous medical treatments is to accelerate the regression in endometrial tissue [17, 31–33]. The effect of hormonal treatment is the prevention of endometriotic tissue proliferation. The general recurrence rate is 30% in patients to whom combined treatment has been applied while this rate is about 35% in the patient group receiving only hormonal treatment [22].

Gonadotropin-releasing hormone (GnRH) agonists and antagonists, progestin, and combined oral contraceptives are listed as the most commonly used medical treatments in endometriosis cases [34, 35]. The utilization of local intrauterine progestin is an effective method with less systematic side effects, and LNG-IUS is the most commonly preferred agent. The expected life of this agent is five years, during which it releases 20 g/day levonorgestrel into the uterine cavity [36]. Theoretically, the side effects of LNG-IUS are less severe since the speed of release and systemic level are lower than stored progestin, subcutaneous implantations, and pure progestin drugs [37–47]. IUS including LNG was first used by Vercellini et al. in 1999 for treatment after endometriosis surgery [41].

LNG-IUS has both local and systemic effects in endometriosis. After the implantation of IUS, LNG-IUS directly releases an overdose of levonorgestrel into the endometrium epithelium. In this way, it prevents the proliferation of endometrial cells by decreasing estrogen and progesterone receptors, and then stromal decidualization and atrophy in endometrial glands are observed [44, 45]. In addition, it has been demonstrated that Fas, a marker of progesterone, estrogen receptors, and apoptosis, was suppressed in endometriosis cases in which LNG-IUS was administered and that this treatment contributed to the antiproliferative effect [46]. In a prospective, noncomparative LNG-IUS study of 334 patients, it was reported that LNG-IUS administration treated the disease stage in 30.8% of the endometriosis cases [47].

Raised CA125 levels often accompany endometriosis and are therefore one of the most researched biomarkers. However, CA125 has low specificity in the diagnosis of endometriosis [48–50].

In the analyses related to the current case, the effects of LNG-IUS and GnRH agents on CA125 were compared in endometriosis cases. According to the data of this study, it was determined that, in the 6th month after the application of LNG-IUS, the CA 125 levels had significantly reduced and there was a positive effect of LNG-IUS on endometriosis lesions [51].

In the current patient, the tumour markers Ca 125: 92.2 (0–35) and Ca 19.9: 138.41 2 (0–37) were determined before treatment. After the application of LNG-IUS, while there was no reduction in the tumour markers at 3 months, when the tumour markers were evaluated at 6 months, they were seen to have receded to normal reference values. At the end of 12 months, the mass had completely recovered and the symptoms had disappeared.

In conclusion, bladder endometriosis should be considered in the differential diagnosis for female patients with bladder masses accompanied by hydronephrosis, who suffer from cyclic painless hematuria attacks. LNG-IUS application may be successfully administered on its own after conservative surgery in patients experiencing moderate and severe pelvic pain due to bladder endometriosis and any other pelvic endometriosis. This treatment method is easily tolerated by the patients since side effects are limited, and the treatment has a positive effect on the patient's quality of life.
